# Two novel quantitative trait linkage analysis statistics based on the posterior probability of linkage: application to the COGA families

**DOI:** 10.1186/1471-2156-6-S1-S121

**Published:** 2005-12-30

**Authors:** Christopher W Bartlett, Veronica J Vieland

**Affiliations:** 1 Center for Statistical Genetics Research, College of Public Health and Roy J. and Lucille A. Carver of Medicine, The University of Iowa, Iowa City, IA, USA; 2 Department of Internal Medicine, The University of Iowa, Iowa City, IA, USA; 3 Department of Psychiatry, Roy J. and Lucille A. Carver of Medicine, The University of Iowa, Iowa City, IA, USA; 4 Program in Public Health Genetics, College of Public Health, The University of Iowa, Iowa City, IA, USA

## Abstract

**Background:**

In this paper we apply two novel quantitative trait linkage statistics based on the posterior probability of linkage (PPL) to chromosome 4 from the GAW 14 COGA dataset. Our approaches are advantageous since they use the full likelihood, use full phenotypic information, do not assume normality at the population level or require population/sample parameter estimates; and like other forms of the PPL, they are specifically tailored to accumulate linkage evidence, either for or against linkage, across multiple sets of heterogeneous data.

**Results:**

The first statistic uses all quantitative trait (QT) information from the pedigree (QT-posterior probability of linkage, PPL); we applied the QT-PPL to the trait ecb21 (resting electroencephalogram). The second statistic allows simultaneous incorporation of dichotomous trait data into the QT analysis via a threshold model (QTT-PPL); we applied the QTT-PPL to combined data on ecb21 and ALDX1. We obtained a QT-PPL of 96% at GABRB1 and a QT-PPL of 18% at FABP2 while the QTT-PPL was 4% and 2% at the same two loci, respectively. By comparison, the variance-components (VC) method, as implemented in SOLAR, yielded multipoint VC LOD scores of 2.05 and 2.21 at GABRB1 and FABP2, respectively; no other VC LODs were greater than 2.

**Conclusion:**

The QTT-PPL was only 4% at GABARB1, which might suggest that the underlying ecb21 gene does not also cause ALDX1, although features of the data complicate interpretation of this result.

## Background

We have developed two new methods for quantitative trait (QT) linkage analysis based on the posterior probability of linkage (PPL) framework [1], which directly measures the probability that a disease gene is linked to a genetic marker or genomic location. The single-locus quantitative trait likelihood as implemented in LIPED is used for analysis, with the trait parameters (allele frequency, genotypic means, and variances) integrated out. This framework has several advantages over pair-wise identity-by-descent (IBD) sharing-based methods: it is based on the full likelihood, uses full phenotypic information, is applicable to pedigrees of arbitrary size and complexity, does not assume normality at the population level or require population/sample parameter estimates; and like other forms of the PPL, it is specifically tailored to accumulate evidence, either for or against linkage, across multiple sets of heterogeneous data. Evidence for linkage is measured on the probability scale (0, 1), and the small prior probability of linkage (2%) is incorporated into the calculation.

These methods were applied to chromosome 4 of the Collaborative Study on the Genetics of Alcoholism (COGA) data using the quantitative ecb21 phenotype, and the dichotomous phenotype ALDX1. ecb21 was chosen because it had yielded a variance components (VC) LOD score of 5.01 near GABRB1 in an analysis using the full set of COGA families [2].

## Methods

### Families and phenotypes

Analysis was performed on all 143 COGA families; average family size was 11.3 (range 5 to 32) and average generations was 2.8 (range 2 to 5). Two pedigrees contained loops and are therefore complex. The two phenotypes considered were resting electroencephalogram (EEG) beta2 spectral/spatial component (ecb21) and the categorical diagnosis of alcoholism (ALDX1). ALDX1 contained two additional categories beyond affected and unaffected which were recoded to unknown for the purpose of analysis. No other changes to phenotypes were made.

### Genetic data

Analysis was conducted on all chromosome 4 markers provided by COGA. Allele frequencies were required all of the analyses presented; we used the values provided with the data, which were estimated by the maximum likelihood method. Map positions were taken as given in the associated map file.

### Statistical analysis

VC analysis was conducted with SOLAR [3]. Analysis was performed with the mean and variance fixed at the founder mean and variance as an approximate multiplex ascertainment correction. There was no transformation of the data nor were any covariates included in the model in order to closely resemble previous analysis of the ecb21 phenotype [2].

The PPL is defined as the integral over [0...1/2) of the posterior density of the recombination fraction θ, computed with the prior probability of linkage set to 2% [4], and a continuous prior on θ over values < 0.50 [5]. The posterior density of θ is calculated as the integral over the trait parameter space of the heterogeneity LOD score [1,6]. Then the PPL is



where π_L _is the prior probability of linkage, **G **is the genotypic data, **X **is the trait data, g() is the prior distribution function for the given parameter, and t is the vector ot trait parameters (allele frequency, penetrances). We include α, the admixture parameter in the QT-PPL to better approximate a multilocus likelihood from the single-locus likelihood.

Here we have used LIPED [7] to compute the individual LOD scores over a descretized grid of values for all constituent parameters, using the program MLIP [8], which parallelizes coverage of the grid space, and was developed by our group for this purpose. Categorical trait PPL analysis was performed as previously described [9,10]. QT-PPL analysis was conducted using the quantitative likelihood implemented in LIPED, which is parameterized in terms of allele frequency, three genotypic means, and three genotypic variances; in our analyses we also allowed for admixture [11]. For computational convenience we restricted the three variances to be equal to one another, which, in our experience developing this method, will not greatly affect the final PPL value and improves computation time (data not shown). Because the QT-PPL (and its derivative below) is based on the same likelihood formulation as the categorical LOD score, it is expected to inherit the same properties (e.g., robustness to modest parameter misspecification, etc.) [12-16]. Results for all PPL analyses in this paper are based on 2-point linkage analysis.

The threshold quantitative trait PPL (QTT-PPL) assumes that all individuals who are affected (in this case, according to the definition of ALDX1) are below some unknown threshold for the underlying quantitative trait (in this case, ecb21). For affected individuals, the cumulative t-distribution (30 df) is used to generate the factors *P*(*x*_*i*_|*g*_*i*_) required by the likelihood, where *x*_*i *_is *i*^th ^person's phenotypic value and *g*_*i *_is their corresponding latent trait genotype. All other subjects are assigned their quantitative (ecb21) trait values, with these same factors calculated using the density *f(x) *= *P(X *= *x*_*i*_|μ_*j*_,*)*, for the t-distribution as before, and where *j *indexes the specific trait genotypic distribution. Here we use the t-distribution instead of the normal density for computational reasons involving the difficulty of estimating probabilities in the extreme tails of the normal distribution. From our experiences in developing this method, this substitution is not expected to have substantive effects on the reported results (data not shown).

The QTT-PPL can be applied when a clinical diagnosis is available for some subjects for whom quantitative measures are not available, yet a relationship between the affection status and the quantitative trait is postulated. But it can also be used to investigate the underlying relationship between the QT and the clinical phenotype by contrasting results from categorical, QT, and QTT analyses because the latter assumes both of the former are related by a common trait locus.

## Results

**Figure 1:**
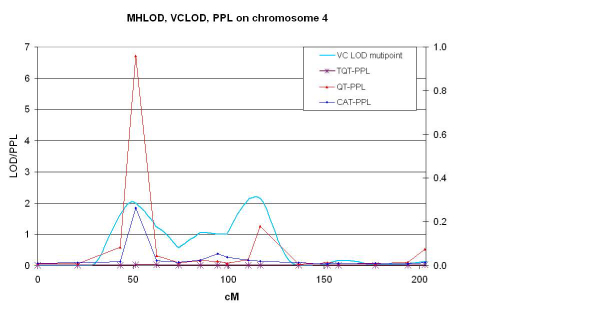
**Summary of PPL and VC analyses**. The LOD scale is on the left and the probability scale is on the right, however there is no direct way to equate values from the 2 scales. Note that PPL values are based on 2-point likelihoods. The data points are connected only for ease of reading.

Two regions are identified for possible follow-up genotyping. The QT-PPL was 96% and 18% at GABRB1 (51 cM) and FABP2 (116 cM), respectively. Categorical PPL analysis gave rise to lower PPLs, 26% and 2%, at these same loci. Multipoint VC analysis in these regions yielded VC LOD scores of 2.05 (2-point, 1.54) and 2.21 (2-point, 2.75). Figure 1 summarizes the results from VC, QT-PPL, and categorical PPL analysis of the entire chromosome. The QTT-PPL yielded a probability of only 4% at GABRB1, far below the QT-PPL of 96%, and below the categorical PPL of 26% at the same location. We note that interpreting the relative magnitudes of the three different PPL statistics in comparison with one another is complicated by features of the data. There were 149 persons who were categorized as affected, but did not have a corresponding QT value. Further, there were 341 persons categorized as unknown for the dichotomous trait who did have a corresponding QT value. Hence, the difference in quantity and pattern of available phenotypic information between the categorical, QT, and QTT analyses was not trivial: the QT analysis used 12% (*n *= 192) more phenotypic information than the categorical analysis, while the QTT-PPL used 30% (*n *= 490) more phenotypic information. These large changes in the number of phenotypes, as well as who in the pedigree was phenotyped, might alone account for the large difference in the threshold QT PPL compared with the categorical and QT analyses. Alternatively, the low QTT-PPL might be indicating a lack of an underlying biological relationship between ecb21 and alcoholism as defined using ALDX1.

## Conclusion

This paper indicates strong evidence for linkage of ecb21 to the GABRB1 region of chromosome 4. This result confirms a previous genome scan using this phenotype in an extended set of the COGA families, which yielded a VC LOD of 5.01 in this same region [2]. The current COGA dataset differs from that of Porjesz et al. [2] in several key ways, particularly, in the available genotyped markers and in sample size. It therefore not surprising that our VC analysis gave differing results from theirs, though there was still some evidence for linkage in the present data based on VC analysis.

However, there has been no equivalent indication of linkage to GABRB1 with a categorical alcoholism phenotype in the literature, while our results indicate a 26% of linkage to alcoholism. When we applied a unified threshold analysis of the categorical and QT phenotypes, implicitly assuming a relationship mediated by the QT, the PPL was only 4%, which is larger than the prior probability of 2%, but not appreciably so. Because the threshold analysis used the largest amount of phenotypic information of all the PPL analyses, we may conclude that it represents a solution closest to the correct assessment of the data; either the relationship of ecb21 phenotype to alcoholism is weak (perhaps non-existent) in this dataset or the relationship of ecb21 to alcoholism departs substantially from the assumed model of the QTT-PPL. The former conclusion is supported by the lack of a categorical linkage of GABRB1 to the alcoholism diagnosis in the literature.

While issues of scale preclude a direct comparison between VC- and PPL-based methods, *prima facie*, it appears that the QT-PPL provided more compelling evidence for linkage than VC analysis of the GAW data. Because all PPL values are on the probability scale (analogous to the chance of rain in a weather forecast), a probability of 96% is a very strong indication that a gene for ecb21 is near GABRB1 even after considering 3 separate analyses of these same data. We are in the process of systematically examining the properties of the QT-PPL and threshold QT-PPL under a variety of single- and multi-locus QTL models, as well as implementing multipoint versions of both statistics. The result of such systematic evaluations will aid in interpretation of the PPL compared to other commonly used linkage methods.

## Abbreviations

COGA: Collaborative Studies on the Genetics of Alcoholism

EEG: Electroencephalogram

GAW: Genetic Analysis Workshop

IBD: Identity by descent

PPL: Posterior probability of linkage

QT: Quantitative trait

QT-PPL: Quantitative trait posterior probability of linkage

QTT-PPL: Threshold quantitative trait posterior probability of linkage

VC: Variance components

## Authors' contributions

Both authors were fully involved in all aspects of this collaborative research endeavor. Both authors read and approved the final manuscript.
